# Structural Engineering of Bimetallic CoCe-ZIF Derives Catalysts with Optimized Electronic Structure for Enhanced Oxygen Electrocatalysis

**DOI:** 10.3390/ma18102251

**Published:** 2025-05-13

**Authors:** Linxiang Zhou, Chaoyang Shi, Huaqi Wang, Danyang Wei, Haodong Jin, Haoqi Li, Zhiwei Meng, Mingli Xu

**Affiliations:** 1 Faculty of Metallurgical and Energy Engineering, Kunming University of Science and Technology, Kunming 650093, China; 20222202009@stu.kust.edu.cn (L.Z.); 20221102003@stu.kust.edu.cn (C.S.); 20202202141@stu.kust.edu.cn (H.W.); weidanyang@stu.kust.edu.cn (D.W.); 20222202173@stu.kust.edu.cn (H.J.); 20222102058@stu.kust.edu.cn (H.L.); 20222102016@stu.kust.edu.cn (Z.M.); 2 National and Local Joint Engineering Research Center for Lithium-ion Batteries and Materials Preparation Technology, Key Laboratory of Advanced Battery Materials of Yunnan Province, Kunming 650093, China

**Keywords:** MOF materials, CoCe bimetal, oxygen electrocatalysis, GDE half-cell test, zinc–air battery

## Abstract

Developing efficient and durable non-precious metal catalysts for oxygen electrocatalysis in fuel cells and zinc–air batteries remains an urgent issue to be addressed. Herein, a bimetallic CoCe-NC catalyst is synthesized through pyrolysis of Co/Ce co-doped metal–organic frameworks (MOFs), retaining the inherently high surface area of MOFs to maximize the exposure of Co-N and Ce-N active sites. The electronic interaction between Co and Ce atoms effectively modulates the adsorption/desorption behavior of oxygen-containing intermediates, thereby enhancing intrinsic catalytic activity. In alkaline media, the CoCe-NC catalyst exhibits E_1/2_ = 0.854 V electrocatalytic capability comparable to commercial Pt/C, along with superior methanol resistance and durability. Notably, CoCe-NC demonstrates an overpotential 84 mV lower than Pt/C at 300 mA cm^−2^ in a GDE half-cell. When the catalyst is employed as a cathode in zinc–air batteries, it demonstrates an open-circuit voltage of 1.47 V, a peak power density of 202 mW cm^−2^, and exceptional cycling durability.

## 1. Introduction

Due to the growing fallout from the energy crisis that stems from traditional petroleum resources becoming increasingly scarce, the task of developing new energy conversion devices is particularly urgent [1,2]. Fuel cells and zinc–air batteries have garnered extensive research attention as new energy devices in the new era [3,4]. However, the oxygen reduction reaction (ORR) located at the cathode is still kinetically slow, which severely limits the widespread use of new batteries [5,6,7]. Although noble metal elements such as Pt, Ir, and Ru can effectively enhance the electrode reaction rate, the scarcity, high cost, and insufficient catalytic stability of noble metals remain challenging problems that need to be addressed urgently [8,9,10,11]. Therefore, the preparation of catalysts for transition metal-based materials with excellent catalytic activity and long-lasting stability has become a priority.

In non-precious metal catalysts studied so far, metal–nitrogen–carbon (M-N-C) materials have attracted attention because of their tunable structures and outstanding performance [12,13]. This is a class of composites consisting of metals, nitrogen, and carbon elements, which has generated intense debate in the field of ORR catalytic [13]. Typically, the metal centers employ transition metals, such as Fe, Co, and Mn, which can exist in the material as single atoms, metal clusters, or nanoparticles, forming highly exposed active sites that confer efficient catalytic activity to the catalysts [14,15,16,17,18]. With the introduction of nitrogen atoms, nitrogen directly coordinates with the metal centers, effectively modulating the electron cloud structure of the metal center, thus significantly enhancing the catalytic ability of the catalyst [19,20,21]. As supporting material, carbon substrates typically possess a high specific surface area and excellent electron transfer capability and stability [22]. Carbon not only provides structural support for metals and nitrogen but also actively influences the catalytic process by enhancing reactant adsorption capacity and electron transfer efficiency [23]. This unique structure allows M-N-C to exhibit a number of outstanding advantages in the ORR field, demonstrating exceptional application prospects in ORR research.

Of the many catalysts, Fe-N-C is being worked on more extensively, owing to the Fe^2+^/Fe^3+^ redox couple present in Fe atoms [24,25]. Fe^2+^ can combine with oxygen molecules to form initial reaction intermediates, while Fe^3+^ absorbs electrons by being reduced, further driving the progression of the ORR and lowering the reaction energy barrier [24,25]. However, the Fe catalytic centers favor 2e⁻ oxygen reduction (producing H_2_O_2_) over the 4e⁻ pathway, leading to efficiency loss in fuel cells [26]. As the most abundant rare earth element on Earth, Ce has attracted significant research interest in recent years due to its substantial reserves and notable cost-effectiveness [27]. Recent studies have shown that Ce atoms also have excellent ORR catalytic activity as metal catalytic activity centers [27,28,29]. This is primarily attributed to the reversible redox couple of Ce^3+^/Ce^4+^ in Ce atoms [30]. The reversibility of the Ce^3+^/Ce^4+^ redox process allows Ce to stably and alternately participate in the ORR, dynamically absorbing and releasing electrons to interact with oxygen-containing intermediates, thereby demonstrating good electrocatalytic activity [28,30]. Furthermore, compared to Fe atoms, Ce atoms do not undergo the Fenton reaction with H_2_O_2_, making them highly compatible with fuel cell devices. Therefore, Ce holds vast potential for application in the field of fuel cell catalysts.

Bimetallic catalysts have garnered significant attention as efficient oxygen electrocatalysts, owing to the synergistic catalytic effects enabled by their dual metallic active sites during reaction processes [31]. Furthermore, the presence of multiple active sites within these catalysts facilitates diverse oxygen electrocatalytic reactions, making bimetallic catalysts a prominent research focus for high-performance zinc–air battery applications. Given the promising ORR potential of Ce atoms, constructing M-Ce (where M represents Fe, Co, Mn, etc.) bimetallic catalysts has emerged as a key research objective [32,33]. Notably, cobalt-based catalysts stand out as promising candidates due to their exceptional chemical stability under alkaline conditions. Consequently, the development of high-efficiency Co-Ce bimetallic oxygen electrocatalysts constitutes the primary research emphasis of this work.

In recent years, metal–organic frameworks (MOFs) have found widespread application in the field of ORR catalysts owing to their multiple advantages, such as large specific surface area, tunable metal centers, organic ligand structures, good electrical conductivity, and stability [34,35,36]. MOFs possess extremely high specific surface areas, exposing more active sites and providing ample opportunities for catalytic reactions [37]. Furthermore, the pore sizes and shapes of MOFs can be precisely designed, and this porosity allows reactants to quickly diffuse to the catalyst surface and interact effectively with active sites, thereby significantly accelerating the reaction rate [38]. This characteristic is particularly advantageous in gas-phase catalytic reactions, effectively enhancing reaction efficiency. More importantly, the metal centers of MOFs are highly tunable. By selecting different metal ions (such as Co, Ni, Fe, Cu, etc.) as nodes, the oxygen reduction performance of MOFs can be flexibly adjusted to suit different reaction environments, exhibiting varied catalytic activities [39]. This structural tunability allows MOFs to optimize their active centers according to specific ORR requirements, thereby enhancing catalytic activity and selectivity. Through the rational design and modification of MOFs, their long-term stability and durability in ORRs can also be enhanced.

Given the outstanding electrochemical properties of Ce and the advantageous application conditions of MOFs in ORR catalysis, this work successfully synthesized CoCe-ZIF MOF material by introducing Zn^2+^, Co^2+^, and Ce^3+^ ions and polymerizing them with dimethylimidazole. Subsequently, the zinc ions were removed from the precursor through high-temperature calcination, ultimately yielding the CoCe-NC catalyst. The CoCe-NC catalyst retains the structural characteristics of a regular dodecahedron, and its enormous specific surface area fully exposes the Co-N and Ce-N active sites, significantly enhancing the catalytic performance of the material.

In 0.1 M KOH, the CoCe-NC catalyst exhibits high electrocatalytic performance (E_1/2_ = 0.854 V), comparable to Pt/C catalysts (E_1/2_ = 0.847 V). Furthermore, after an electrocatalytic stability test, the performance of the CoCe-NC catalyst remains essentially at its initial level. In GDE half-cell tests, the overpotential of the CoCe-NC is 84 mV lower than that of the commercial Pt/C at 300 mA cm^−2^, and it exhibits smaller electronic transfer impedance and superior stability. In applications in zinc–air batteries, the CoCe-NC catalyst demonstrates an OCV of 1.47 V and the power density of 202 mW cm^−2^, and it still exhibits high stability after 300,000 s of cycling tests.

## 2. Experimental Section

### 2.1. Chemicals

Co(NO_3_)_2_·6H_2_O, 99%, Aladdin (Shanghai, China), Ce(NO_3_)_3_·6H_2_O, 99.5%, Aladdin, Zn(NO_3_)_2_·6H_2_O, 99%, Aladdin, 2-methylimidazole, 98%, Aladdin, CH_3_OH, 99.5%, Aladdin, EtOH, 99.7%, Tianjin Fuyu Chemical (Tianjin, China), H_2_SO_4_, 98%, Tianjin Fengchuan Chemical (Tianjin, China), KOH, 95%, Macklin, Zn(CH_3_COO)_2_, 99%, Macklin, Nafion 5 wt%, DuPont (Wilmington, DE, USA), Pt/C 20 wt% JM, and RuO_2_ (Ru > 75%, Sunero, Woolloongabba, Australia).

For all solutions, 18.25 MΩ ultrapure water was used.

### 2.2. Material Synthesis

Synthesis of CoCe-NC: Dissolve 2.32 g of Zn(NO_3_)_2_·6H_2_O, 1.09 g of Co(NO_3_)_2_·6H_2_O, and 0.36 g of Ce(NO_3_)_3_·6H_2_O in 60 mL of methanol, and ultrasonicate the mixture to achieve homogeneous dispersion, obtaining a metal salt precursor solution. Simultaneously, dissolve 2.464 g of 2-methylimidazole in 60 mL of methanol and ultrasonicate to form a homogeneous solution. Under stirring, slowly pour the metal salt precursor solution into the 2-methylimidazole solution and continue stirring for 6 h. Centrifuge the mixture to collect the product and then wash it several times with methanol via centrifugation. Dry the product in a vacuum oven at 65 °C to obtain the ZnCoCe-ZIF powder. Finally, carbonize the ZnCoCe-ZIF powder at 900 °C for 2 h under N_2_ atmosphere at a ramp rate of 5 °C per minute to obtain the CoCe-NC catalyst.

When no Ce source is added to the metal precursor solution, the final product is Co-NC; if no Co source is added, the final product is Ce-NC; when neither Ce nor Co source is added, the ZIF-8 precursor is obtained, which is subsequently pyrolyzed to obtain NC.

### 2.3. Material Characterization

XRD spectra were obtained by testing on Cu targets at a scan rate of 5°/min. The specific surface area was calculated using BET surface area testing. Topography and structure were photographed by FE-SEM (ZEISS sigma 300) and TEM (FEI Tecnai G2 F20). XPS experiments were implemented on Al Kα radiation (Thermo Scientific K-Alpha). The content of Co and Ce in all samples was analyzed by ICP-OES (Agilent 5110). Raman analysis required a confocal Raman microscope (CRM) (Alpha 300R, WITec GmbH, Ulm, Germany).

### 2.4. Electrochemical Measurements

A CHI 760E electrochemical workstation was employed to characterize all relevant electrochemical parameters through cyclic voltammetry and electrochemical impedance spectroscopy measurements. The catalysts used a three-electrode system, a Pt sheet as the counter electrode, and an AgCl electrode as the reference electrode, using a catalyst-loaded working electrode (0.6 mg cm^−2^ catalyst film loading for the samples and 0.2 mg cm^−2^ catalyst film loading for the commercial Pt/C).

The reference relationship between the measured potential and the AgCl condition was converted using the following equation:

(1) $$E\;\left(\mathrm{RHE}\right)=E\;(\mathrm{AgCl})+0.0591\mathrm{pH}+0.197\;$$

The kinetic current density was obtained according to the following equation (K-L equation):

(2) $$\frac{1}{j}=\frac{1}{j_{k}}+\frac{1}{j_{L}}=\frac{1}{j_{k}}+\frac{1}{\beta \omega^{0.5}}$$

(3) $$n=4\;\times \;\frac{I_{D}}{\frac{I_{R}}{N}+I_{D}}$$

The n and H_2_O_2_% were obtained by RRDE experiments using the following equations:

(4) $${H_{2}O}_{2}\left(\%\right)=200\;\times \;\frac{\frac{I_{R}}{N}}{\frac{I_{R}}{N}+I_{D}}$$

### 2.5. OER Test

The OER test uses the same three-electrode system at the workstation as the ORR, with the working electrode replaced by a nickel foam containing the catalytic material. The loading of RuO_2_ on nickel foam was 0.25 mg cm^−2^ for the samples and 1.0 mg cm^−2^ for the CoCe-NC.

### 2.6. GDE Half-Cell Test

The GDE half-cell test in this study simulated the practical working environment of AEMFC to test the catalyst at high current density. The GDE test is also a three-electrode operation, but the work electrode is replaced by a catalyst-loaded carbon cloth. The loadings of both the CoCe-NC catalyst and Pt/C were 1 mg cm^−2^, and the electrolyte was 1.0 M KOH.

### 2.7. Zn–Air Battery Test

Zinc–air batteries use zinc flakes as the negative electrode. Electrode sheets were prepared using carbon paper, waterproof, breathable film, and a nickel foam catalyst rolled and pressed together, and the pressed material was used as a cathode after coating the catalyst on the carbon paper. The loading of CoCe-NC was 1.0 mg cm^−2^, and the loading of commercial Pt/C + RuO_2_ was 1.0 mg cm^−2^. Furthermore, 6 M KOH and 0.2 M Zn(Ac)_2_ were used as the electrolyte for testing.

## 3. Results and Discussion

### 3.1. Catalyst Physical Structure Characterization

**Figure 1:**
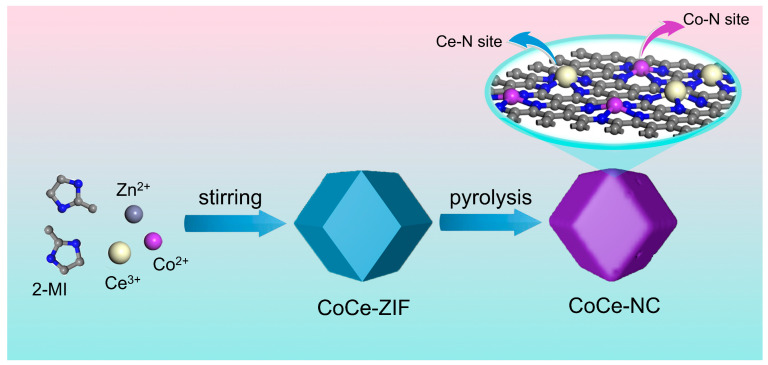
Illustration of the synthesis process of CoCe-NC.

**Figure 2:**
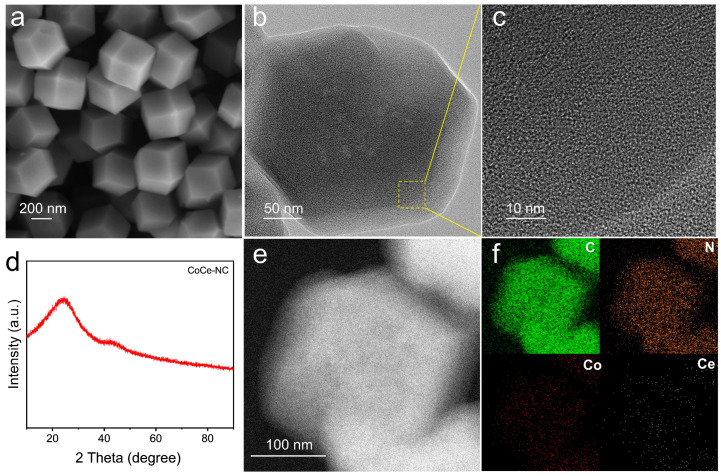
(**a**) FE-SEM image of CoCe-NC. (**b**) TEM image of CoCe-NC. (**c**) HR-TEM image of CoCe-NC. (**d**) XRD pattern of CoCe-NC. (**e**) TEM image of CoCe-NC under the dark field. (**f**) EDS elemental mapping of CoCe-NC.

The preparation of CoCe-NC catalysts is exhibited in Figure 1. Initially, Zn^2+^, Co^2+^, and Ce^3+^ ions are mixed with 2-methylimidazole through stirring to obtain a Co and Ce-doped ZIF precursor. Upon high-temperature treatment at 900 °C, the volatilization of Zn atoms creates a porous three-dimensional framework, while the doped Co and Ce atoms coordinate with nitrogen to form stable Co-N and Ce-N configurations. This process ultimately yields the CoCe-NC catalyst featuring the dual active sites of Co-N and Ce-N coordination structures. XRD spectroscopy analysis of the precursor reveals that it possesses a standard MOF precursor structure (Figure S1). The morphology captured by FE-SEM is shown in Figure 2a, indicating that the prepared CoCe-NC retains the regular dodecahedral structure of Co-NC and NC (Figure S2). This three-dimensional structure ensures the transportation of oxygenated intermediates and provides more reaction sites and expanded reaction space for the ORR, thereby enhancing catalytic efficiency. Figure 2b presents the TEM image analysis of CoCe-NC, revealing no significant metal aggregation or formation of metal particles, indicating that the metals are highly dispersed within the graphite layers. This highly dispersed structure fully exposes the active sites, maximizing the utilization of each site and effectively improving catalytic activity. HR-TEM images show a typical amorphous structure with randomly oriented graphitic carbon (Figure 2c). This structure effectively expands the active area, providing assurance for hosting abundant catalytic sites.

The XRD spectra shown in Figure 2d and Figure S3 show only two peaks corresponding to structures (002) and (101) of graphitic carbon, with no observable metal intensity peaks, indicating the absence of metal particle formation, which aligns with the TEM results. The mapping results for corresponding elements demonstrate that Co, Ce, and N elements are uniformly distributed throughout the material (Figure 2e,f). ICP-OES is a commonly used technique for quantitative elemental analysis. ICP-OES reveals that the Co and Ce contents in CoCe-NC are 2.32% and 0.09%, respectively, confirming the successful incorporation of Co and Ce elements into the catalytic material (Table S1).

**Figure 3:**
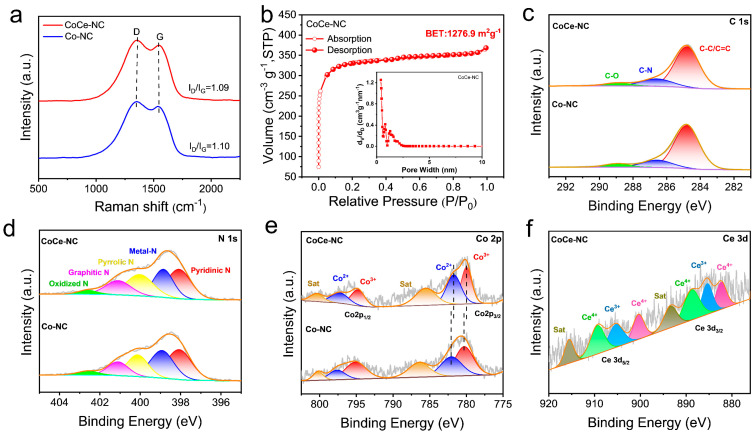
(**a**) Raman patterns of CoCe-NC and Co-NC. (**b**) BET curves of CoCe-NC, inset: pore size distributions. (**c**) C 1s, (**d**) N 1s, (**e**) Co 2p, and (**f**) Ce 3D high-resolution XPS spectra of CoCe-NC and Co-NC.

Raman spectroscopy is also an important characterization technique for analyzing the defects and graphitization of materials. Figure 3a displays the Raman pattern of CoCe-NC and Co-NC catalysts. Both curves clearly exhibit two distinct Raman characteristic peaks: the D-band and the G-band. The D-band represents the amorphous structure, while the G-band signifies sp^2^ hybridization and graphitic ordering [40]. The I_D_/I_G_ is used to evaluate the defect and graphitization degree of the material. The I_D_/I_G_ values for CoCe-NC and Co-NC are 1.09 and 1.10, indicating that the prepared materials both exhibit high graphitization structures, and the addition of Ce does not significantly alter the defects or graphitization degree of the material. A large specific surface area is an indispensable condition for an excellent ORR catalyst, as a larger surface area facilitates the exposure of active sites, thereby significantly enhancing the electro-chemical activity of the catalyst. As shown in Figure 3b and Figure S4, CoCe-NC and Co-NC have specific surface areas of 1276.9 m^2^ g^−1^ and 1160.4 m^2^ g^−1^. Thanks to the three-dimensional structure of the MOF, the enormous specific surface area, combined with highly dispersed metal sites, can substantially enhance the catalytic properties of materials. Furthermore, pore size analysis reveals that the pores in CoCe-NC and Co-NC are predominantly micropores, which contribute to exposing more catalytic sites, laying the foundation for the high electrocatalytic activity of the catalyst.

XPS is widely used to analyze the chemical composition and bonding states of materials. Figure 3c presents the C 1s XPS spectrum, which can be fitted with characteristic peaks of the three carbons, corresponding to C-C/C=C (284.8 eV), C-N (286.6 eV), and C-O (289.0 eV) [41], respectively. This confirms the abundance of C-N structures in the material, while the small amount of O atoms primarily originates from oxygen-containing functional groups. Figure 3d shows the N 1s spectrum, which can be fitted into five characteristic peaks corresponding to pyridinic-N (298.1 eV), metal-N (298.9 eV), pyrrolic-N (400.1 eV), graphitic-N (401.1 eV), and oxidized-N (402.6 eV) [33]. The percentage contents of these five types of N are presented in Table S2. Compared to Co-NC, CoCe-NC has a higher content of metal-N, which may be attributed to the incorporation of Ce, leading to the formation of Ce-N structures. Figure 3e depicts the Co 2p XPS spectrum. In both CoCe-NC and Co-NC, the valence states of Co atoms are Co^3+^ (780.3 eV and 791.1 eV) and Co^2+^ (782.1 eV and 793.6 eV) [42]. However, due to the addition of Ce atoms, the Co 2p peak in CoCe-NC shifts toward lower binding energy compared to Co-NC, indicating that Co atoms gain electrons. The electron-rich Ce atoms enable electron transfer to Co atoms, inducing charge redistribution within the Co species. This adjusts the d-band center of Co to an optimal position, which is beneficial for optimizing the adsorption and desorption of oxygenated intermediates on Co sites, thereby accelerating the ORR process [43]. Figure 3f displays the Ce 3d XPS spectrum, with fitted peaks corresponding to Ce^3^⁺ (885.4 eV and 905.3 eV) and Ce^4^⁺ (882.4 eV, 888.8 eV, 900.4 eV, and 909.4 eV), confirming the successful integration of Ce into the carbon framework [43].

### 3.2. Electrochemical Testing

In order to investigate the ORR electrocatalytic activity of the CoCe-NC catalyst, a series of ORR tests were conducted in a 0.1 M KOH electrolyte. For comparison, Pt/C was used as a reference catalyst. To systematically optimize the Co/Ce ratio, a series of Co_x_Ce_y_-NC catalysts were synthesized by precisely controlling metal precursor inputs (Figure S5). Through comparative analysis of the linear sweep voltammetry (LSV) profiles, the optimal metal loadings were identified as 4 mmol Co and 0.8 mmol Ce.

**Figure 4:**
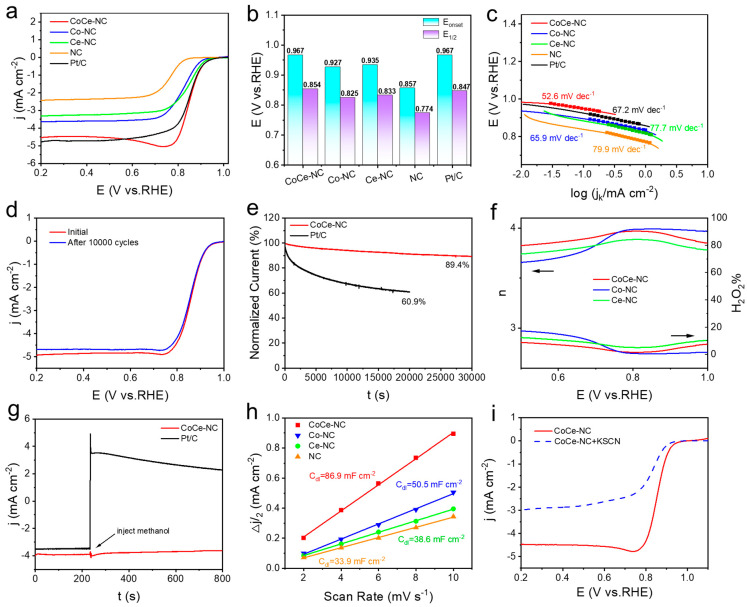
(**a**) ORR LSV curves and (**b**) histograms of E_onset_ and E_1/2_ of corresponding catalysts. (**c**) Tafel plots of various catalysts. (**d**) LSV curves of CoCe-NC before and after 10,000 CV cycles. (**e**) Calculated current test at 0.70 V on CoCe-NC and Pt/C electrode. (**f**) H_2_O_2_ yield and electron transfer number (n) of various catalysts. (**g**) Calculated current test at 0.7 V on CoCe-NC and Pt/C electrode with injected 3 M methanol. (**h**) Relationship between capacitor current and scan rate. (**i**) LSV curves before and after the addition of 10 mmol KSCN.

Figure 4a presents the ORR LSV polarization curves, with the results summarized in Figure 4b and Table S3. CoCe-NC exhibits the best reactive (E_onset_ = 0.967 V, and E_1/2_ = 0.854 V), matching the performance of Pt/C (E_onset_ = 0.967 V, and E_1/2_ = 0.847 V). This indicates that CoCe-NC is comparable to Pt/C in the field of ORR catalysis, providing a performance basis for its practical applications. Also, we compared the E_onset_ and E_1/2_ of CoCe-NC catalysts with those of recent Co-based catalysts and found that their ORR catalytic performance is at the forefront of research (Table S4). Figure 4c shows the Tafel slope curves of the catalysts. The Tafel slope reflects the kinetic rate, with a smaller slope indicating a faster catalytic rate. The Tafel slope of CoCe-NC is only 52.6 mV dec^−1^, suggesting that it has the fastest reaction rate. The normalization of current density to kinetic current density for intrinsic activity evaluation shows that CoCe-NC achieves the biggest values, indicating exceptional kinetic performance. (Figure S6).

In the process of catalyst usage, the lifespan of the catalyst is equally an inevitable key issue. We employ Accelerated Durability Tests (ADTs) and chronoamperometric tests to verify the stability. After 10,000 CV cycles, the E_onset_ and E_1/2_ of CoCe-NC remain basically unchanged (Figure 4d), whereas the E_1/2_ of Pt/C decreases by 16 mV after 10,000 cycles of CV (Figure S7). Figure 4e shows that after 30,000 s testing, CoCe-NC retains 89.4% of its initial current density, significantly outperforming Pt/C (60.9% retention at 20,000 s). These results indicate that CoCe-NC exhibits impressive stability, laying a foundation for its application in fuel cells and zinc–air batteries.

In addition, the types of ORRs were also investigated. ORRs can be categorized into two-electron and four-electron pathways. The two-electron pathway produces 100% H_2_O_2_, and the four-electron pathway completely reduces O_2_ to H_2_O, with a theoretical hydrogen peroxide production rate of 0% [44]. For membrane fuel cells, the generation of hydrogen peroxide species can corrode the polymer membrane, leading to reduced battery lifespan. Therefore, the four-electron reaction pathway is optimal for the battery. The H_2_O_2_ yield and the average number of electron transfers of the catalyst can be measured by rotating ring disk electrode (RRDE) to obtain the results. The results show that CoCe-NC exhibited the most ideal performance, with an average H_2_O_2_ production rate below 10% and an average number of electron transfers above 3.8, indicating that its catalyzed ORR predominantly follows the four-electron pathway (Figure 4f). Furthermore, calculations using the K-L equation also confirm that the ORR catalyzed by CoCe-NC is primarily a four-electron reaction (Figure S8).

Similarly, methanol crossover tolerance is also a critical issue in the use of fuel cells. The test results shown in the figure indicate that the current of CoCe-NC showed only slight fluctuation after methanol addition and quickly recovered to the initial value compared to commercial Pt/C, which suggests that the CoCe-NC catalyst has excellent methanol tolerance (Figure 4g).

The enhanced electrochemically active surface area (ECSA) of the catalyst correlates with increased exposure to active sites, a critical feature for high-performance ORR catalysis. This ECSA exhibits a direct proportionality to double-layer capacitance (C_dl_), as evidenced by CV measurements in non-faradaic regions across multiple scan rates (Figure S9). CoCe-NC has the largest C_dl_ value, indicating that it possesses the largest ECAS (Figure 4h). To explore the type of active sites in CoCe-NC, poisoning with KSCN is performed, and it is found that the reactivity of the catalyst is significantly reduced. This is due to the metal sites being covered by SCN^-^, directly proving that the active sites in the catalyst are the metal sites (Figure 4i). Similarly, we explored the ORR catalytic activity of CoCe-NC under an acidic electrolyte (0.5 M H_2_SO_4_). As shown in the LSV polarization curves (Figure S10), CoCe-NC still exhibits a performance gap compared to commercial Pt/C catalysts.

For zinc–air batteries, the oxygen evolution reaction (OER) is equally crucial as an electrode reaction. Therefore, we tested it on a nickel foam substrate in 1 M KOH, with RuO_2_ used as a comparison for exploration. Figure S11a shows the OER LSV polarization curves of the catalysts. At 50 mA cm⁻^2^ current density, CoCe-NC demonstrates a 370 mV overpotential, outperforming RuO_2_, which requires 430 mV under identical conditions (Figure S11b). Figure S11c presents the fitted Tafel slopes for the OER of the catalysts, indicating that CoCe-NC has a smaller Tafel slope, which signifies its optimal OER kinetic rate. Electrochemical impedance spectroscopy (EIS) was also employed to further evaluate the charge transfer resistance of the catalysts for OER. As shown in Figure S11d, CoCe-NC exhibits the smallest charge transfer resistance. These results indicate that the CoCe-NC catalyst also exhibits impressive performance in the field of OERs and holds promising application prospects in areas such as zinc–air batteries.

### 3.3. GDE Half-Cell Testing

**Figure 5:**
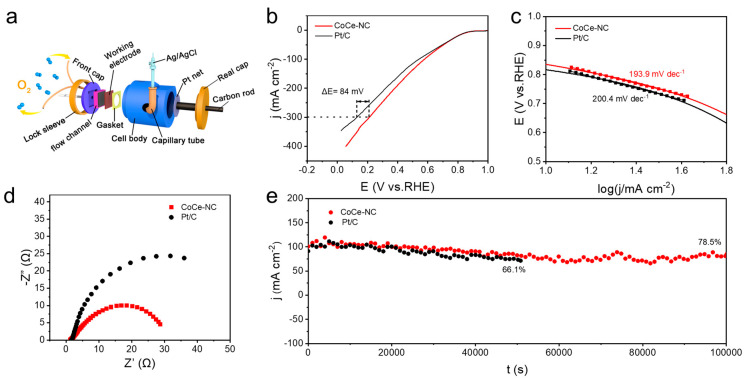
(**a**) Schematic diagram of half-cell [40]. (**b**) LSV polarization curves of CoCe-NC and Pt/C using half-cell. (**c**) Tafel plots. (**d**) Nyquist plots of CoCe-NC and Pt/C at 0.7 V. (**e**) i-t test of CoCe-NC and Pt/C.

To explore the actual operational performance of catalysts, we used a gas diffusion electrode (GDE) device to test the catalysts under conditions that simulate fuel cell applications. Figure 5a illustrates the configuration of the GDE half-cell. The catalyst was loaded on a gas diffusion layer to be used as the cathode in the device, with an Ag/AgCl electrode connected to a capillary tube to be used as a reference electrode, and a Pt grid was connected to a carbon rod to be used as a counter electrode. The gas flow channel provides sufficient oxygen to the air cathode, and the oxygen fully contacts the catalyst on the working electrode as it passes through the graphite gas flow channel, significantly reducing mass transfer resistance and minimizing the mass transfer resistance, thereby enabling the achievement of current densities hundreds of times higher than those obtained with an RDE (rotating disk electrode) and more rapidly reflecting the activity of the catalyst in the fuel cell under actual operating conditions [40]. The LSV polarization curve measured in this setup is shown in Figure 5b, where CoCe-NC exhibits a more pronounced performance advantage over commercial Pt/C. At a high current density of 300 mA cm⁻^2^, the overpotential of CoCe-NC is 84 mV lower than that of commercial Pt/C. Additionally, the Tafel slope indicates that CoCe-NC has superior kinetic performance compared to Pt/C (Figure 5c).

With the electrochemical impedance diagram (Figure 5d), it can be observed that both CoCe-NC and commercial Pt/C exhibit only one arc, indicating that the ORR process for both catalysts is primarily affected by kinetics. Furthermore, the resistance of CoCe-NC is significantly lower than that of Pt/C, which indicates that the charge transfer resistance of the catalyst is also small at high current densities. This will facilitate the catalyst for effective ORR in real fuel cell application scenarios. The catalyst stability under high current density conditions was evaluated through chronoamperometric measurement (Figure 5e). Remarkably, the CoCe-NC catalyst demonstrated superior durability by retaining 78.5% of its initial current density after an extended 100,000 s operation at 100 mA cm⁻^2^, while the Pt/C counterpart showed significant performance degradation with only 66.1% retention after a shorter 50,000 s test period under identical current density conditions. These results demonstrate that CoCe-NC exhibits impressive catalytic performance compared to commercial Pt/C under high current density conditions relevant to practical fuel cells, providing substantial insights for the future application of CoCe-NC in real-world fuel cell systems.

### 3.4. Zinc–Air Battery Testing

**Figure 6:**
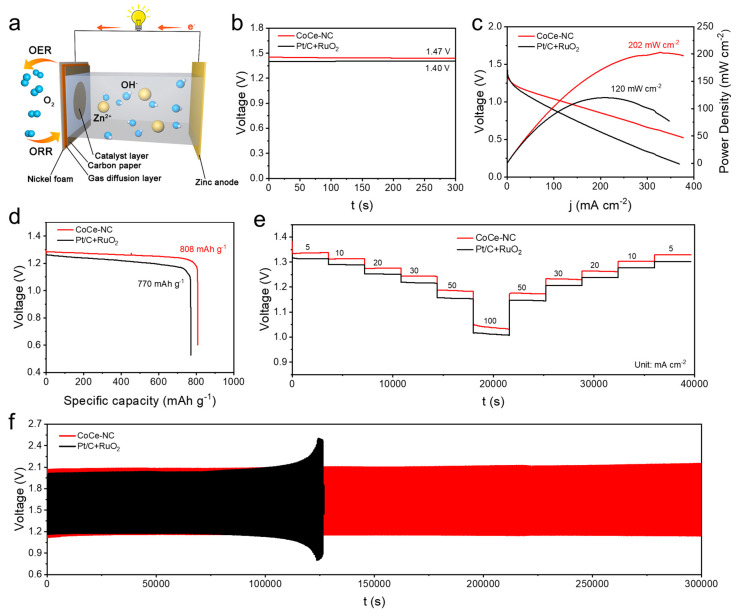
(**a**) Schematic diagram of ZAB battery [40]. (**b**) OCV of batteries. (**c**) Discharge polarization curves and corresponding power density. (**d**) Specific capacity plots at 10 mA cm^−2^. (**e**) Discharge curves at various current densities. (**f**) Galvanostatic charge–discharge cycling curves at 7.8 mA cm^−2^.

Given the catalytic activities of CoCe-NC, we assembled it as the cathode in a zinc–air battery to test its application performance. Meanwhile, commercial Pt/C + RuO_2_ was used as the cathode comparison catalyst, with the installation shown in Figure 6a. The catalyst was loaded onto the cathode, while a polished zinc sheet was used as the anode. Figure 6b shows the open-circuit voltage (OCV) test of the catalysts, where the CoCe-NC-based ZABs exhibit an OCV of 1.47 V, superior to the 1.40 V of Pt/C + RuO_2_, which is also consistent with the measurements from a multimeter (Figure S12). We also conducted peak power density tests on the zinc–air batteries (Figure 6c) and found that the CoCe-NC-based ZABs exhibit a high peak power density of 202 mW cm^−2^, significantly higher than 120 mW cm^−2^ of Pt/C + RuO_2_, and the peak power density of CoCe-NC ranks among the top in recent research (Table S5). At the same time, the specific discharge capacity of the catalysts was tested under 10 mA cm^−2^ (Figure 6d). The specific discharge capacity of CoCe-NC is 808 mAh g^−1^, while that of Pt/C + RuO_2_ is only 770 mAh g^−1^. Battery stability is an important indicator for measuring battery performance, and in chronopotentiometry measurements at different current densities (Figure 6e), CoCe-NC maintains a high voltage output at high current densities, and as the current density decreases, the discharge voltage is able to recover to its initial value. Similarly, the CoCe-NC-based ZABs maintain essentially unchanged charge–discharge voltages after 300,000 s of charge–discharge cycling, whereas the commercial Pt/C + RuO_2_ lost its charge–discharge capability after 125,000 s of testing (Figure 6f). Both tests demonstrate the excellent stability of the CoCe-NC-based ZABs.

In summary, CoCe-NC exhibits impressive catalytic performance in practical applications of zinc–air batteries, outperforming the commercially available Pt/C + RuO_2_ and providing a valuable reference for replacing precious metal catalysts.

## 4. Conclusions

In this work, we successfully incorporate Co and Ce bimetallic elements into MOF materials to construct a CoCe-NC catalyst enriched with M-N active sites. This catalyst not only retains the high specific surface area of MOF material, allowing for full exposure of Co-N and Ce-N active sites, but also regulates the adsorption/desorption process of oxygen-containing intermediates through electron transfer between Co and Ce atoms, thereby endowing the catalyst with excellent electrocatalytic activity. CoCe-NC exhibits ORR performance (E_onset_ = 0.967 V and E_1/2_ = 0.854 V) comparable to that of Pt/C and demonstrates superior methanol tolerance and stability. In GDE half-cell tests, CoCe-NC maintains catalytic activity and stability superior to commercial Pt/C even at high current densities. Furthermore, when applied in zinc–air batteries, CoCe-NC exhibits an OCV of 1.47 V and peak power density of 202 mW cm⁻^2^, and it maintains stable charge–discharge performance after 300,000 s of charge–discharge cycles. These results indicate that CoCe-NC acts as an impressive bifunctional oxygen electrocatalyst.

## Data Availability

The original contributions presented in this study are included in the article/Supplementary Materials. Further inquiries can be directed to the corresponding author.

## Supplementary Materials

The following supporting information can be downloaded at: https://www.mdpi.com/article/10.3390/ma18102251/s1, SEM image, XRD spectrograms, nitrogen adsorption–desorption isotherm and pore size, CV curves, LSV curves, ECSA-normalized LSV, OER parameters (LSV curves, Tafel slope, and Nyquist plots), and digital images of output circuit voltage. References [45,46,47,48,49,50,51,52,53,54,55,56,57,58,59,60,61,62,63] are cited in the supplementary materials.
