# Supplementary material for: Structural Engineering of Bimetallic CoCe-ZIF Derives Catalysts with Optimized Electronic Structure for Enhanced Oxygen Electrocatalysis
Source: Materials (Basel). 2025 May 13;18(10):2251. doi: 10.3390/ma18102251 (PMC12113102; doi:10.3390/ma18102251)
Supplement: Supplementary file 1 [file materials-18-02251-s001.zip › materials-3587277-supplementary.pdf]

Supporting Information

Structural engineering of bimetallic CoCe-ZIF derives catalysts with the optimized electronic structure for enhanced oxygen electrocatalysis

*Linxiang Zhou[a,b], Chaoyang Shi[a,b], Huaqi Wang[a,b], Danyang Wei[a,b], Haodong Jin[a,b], Haoqi Li[a,b], Zhiwei Meng[a,b], Mingli Xu[a,b]**

[a] *Faculty of Metallurgical and Energy Engineering, Kunming University of Science and Technology, Kunming, 650093, China*

[b] *National and Local Joint Engineering Research Center for Lithium-ion Batteries and Materials Preparation Technology, Key Laboratory of Advanced Battery Materials of Yunnan Province, Kunming 650093, China*

Corresponding author: Mingli Xu: xumingli0326@126.com

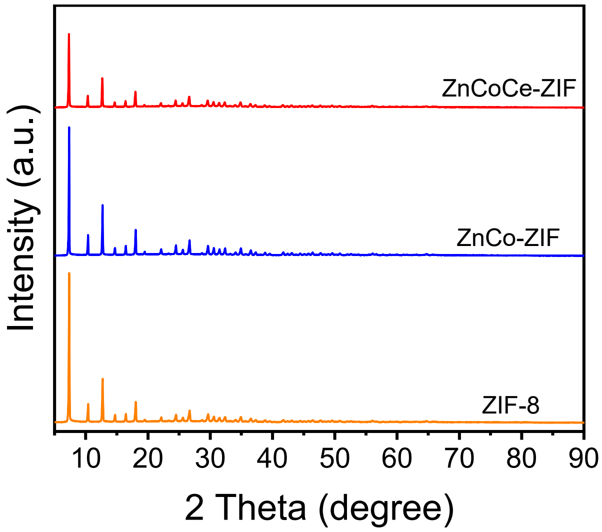

Figure S1. XRD patterns of ZIF-8, ZnCo-ZIF, and ZnCoCe-ZIF.

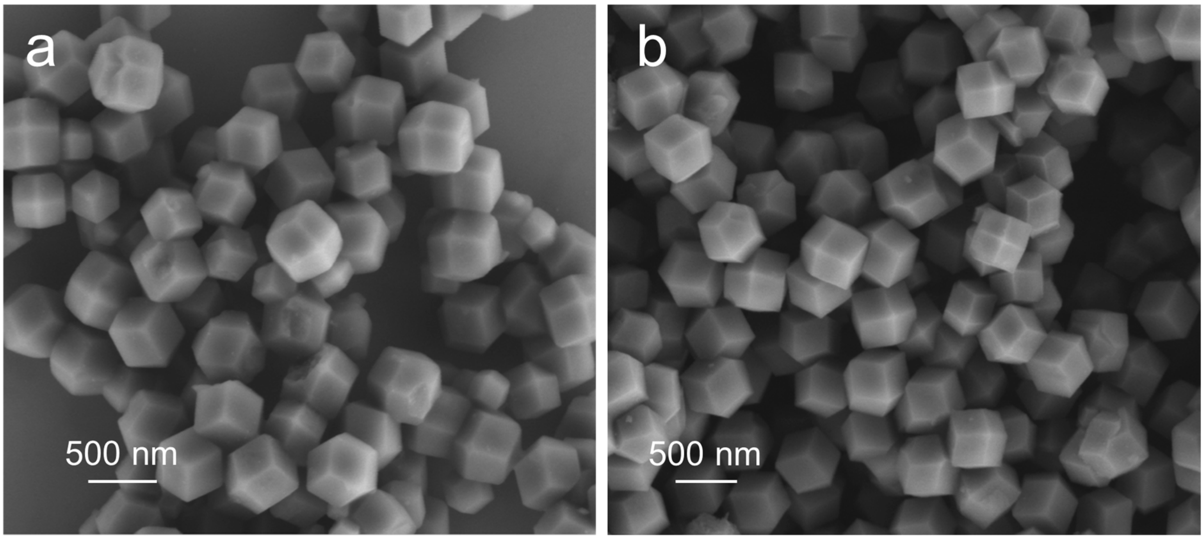

Figure S2. SEM image of (a) Co-NC, (b) NC.

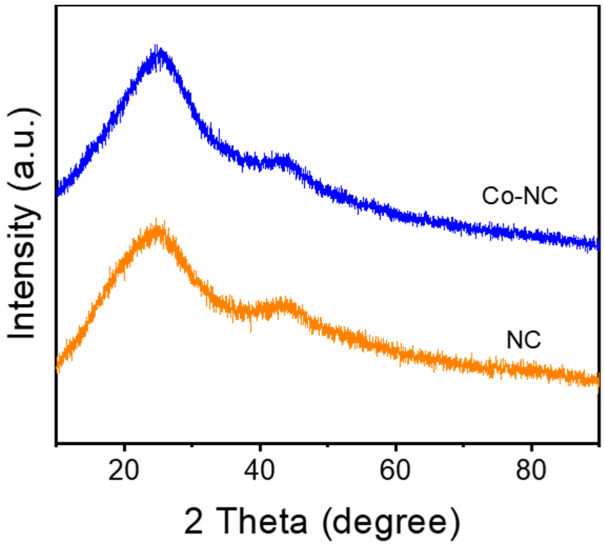

Figure S3. XRD patterns of Co-NC and NC.

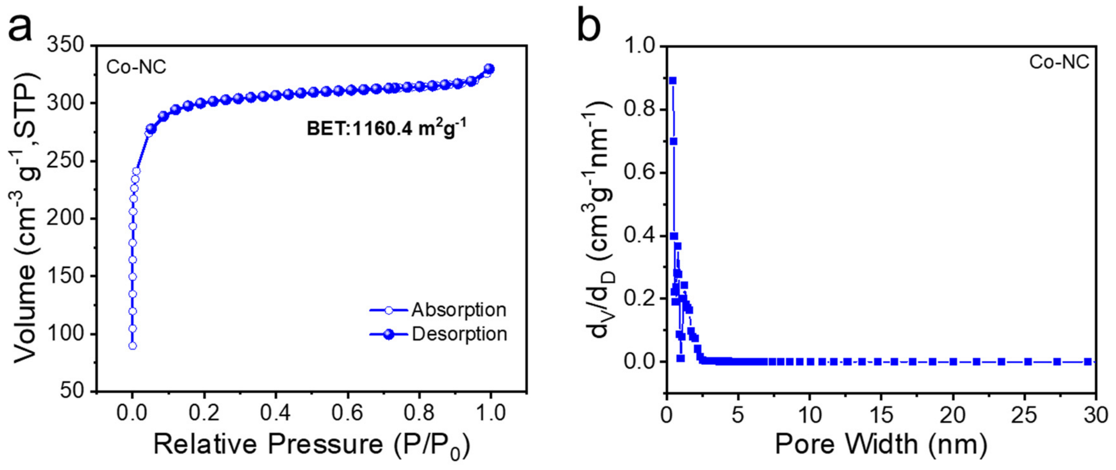

Figure S4. (a) Nitrogen adsorption–desorption isotherm and (b) Pore size distribution of Co-NC.

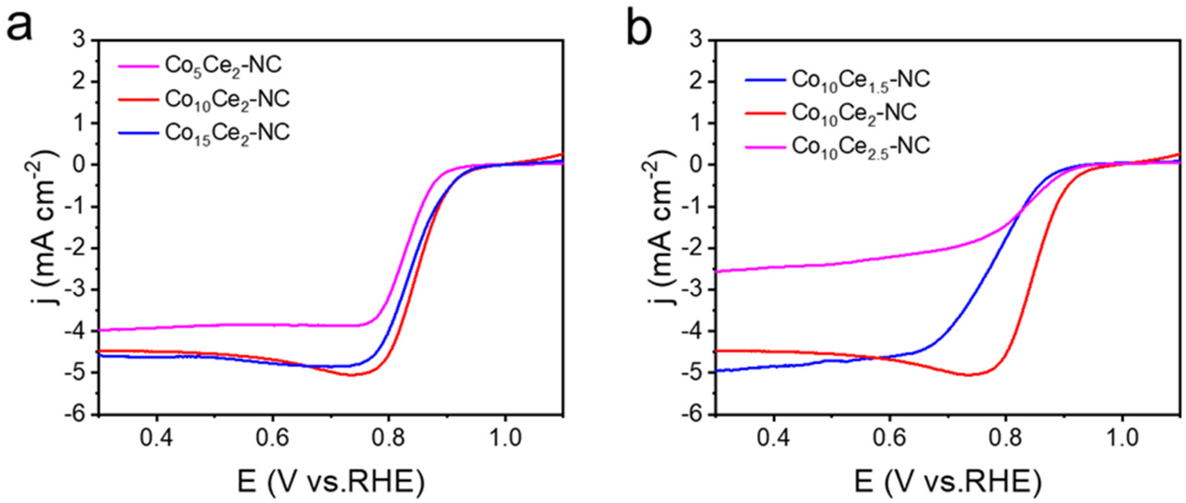

Figure S5. LSV curves of catalysts prepared with different molar ratio additions of Co/Ce (a) adjust the addition ratio of Co, (b)adjust the addition ratio of Ce).

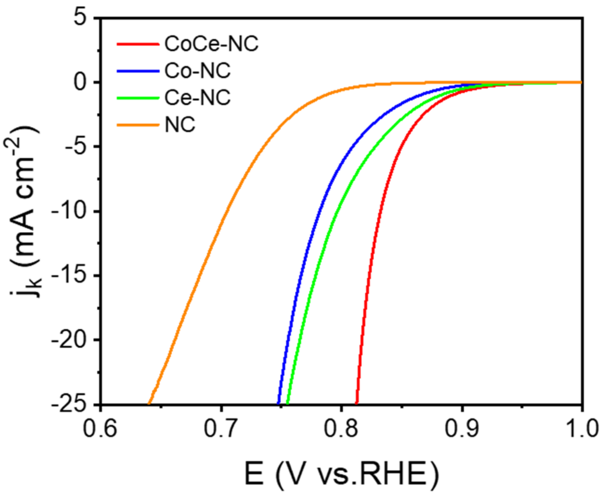

Figure S6. Kinetic current density of catalysis.

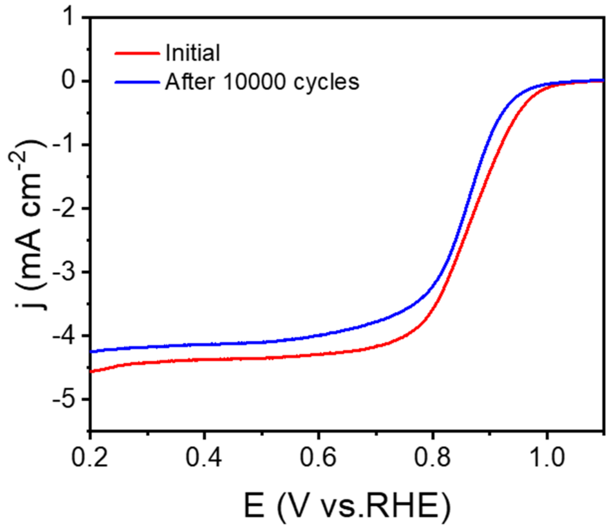

Figure S7. LSV curves of Pt/C and before and after 10,000 CV cycles.

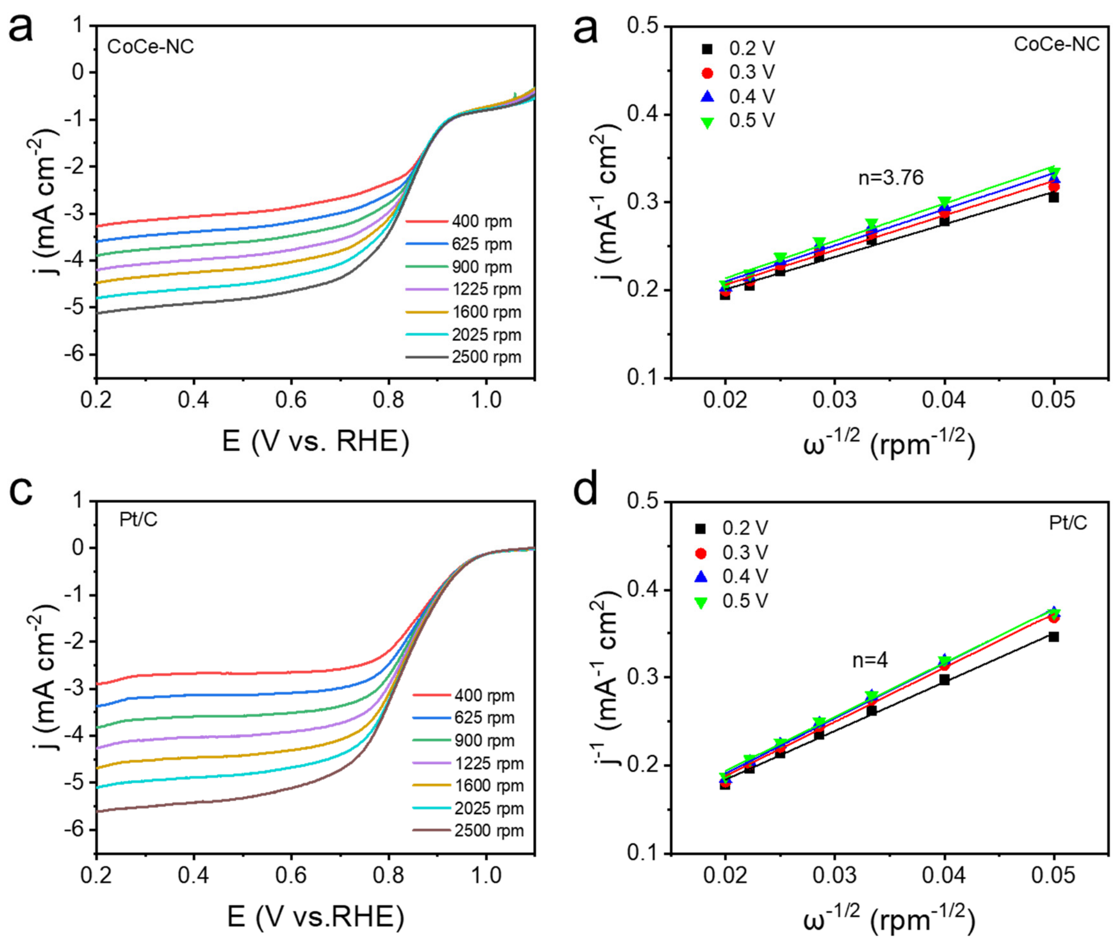

Figure S8. LSV curves of (a) CoCe-NC and (c)Pt/C at 400 - 2500 rpm in O_2-saturated 0.1 M KOH. The (b) CoCe-NC and (d) Pt/C K-L diagram obtained through LSV.

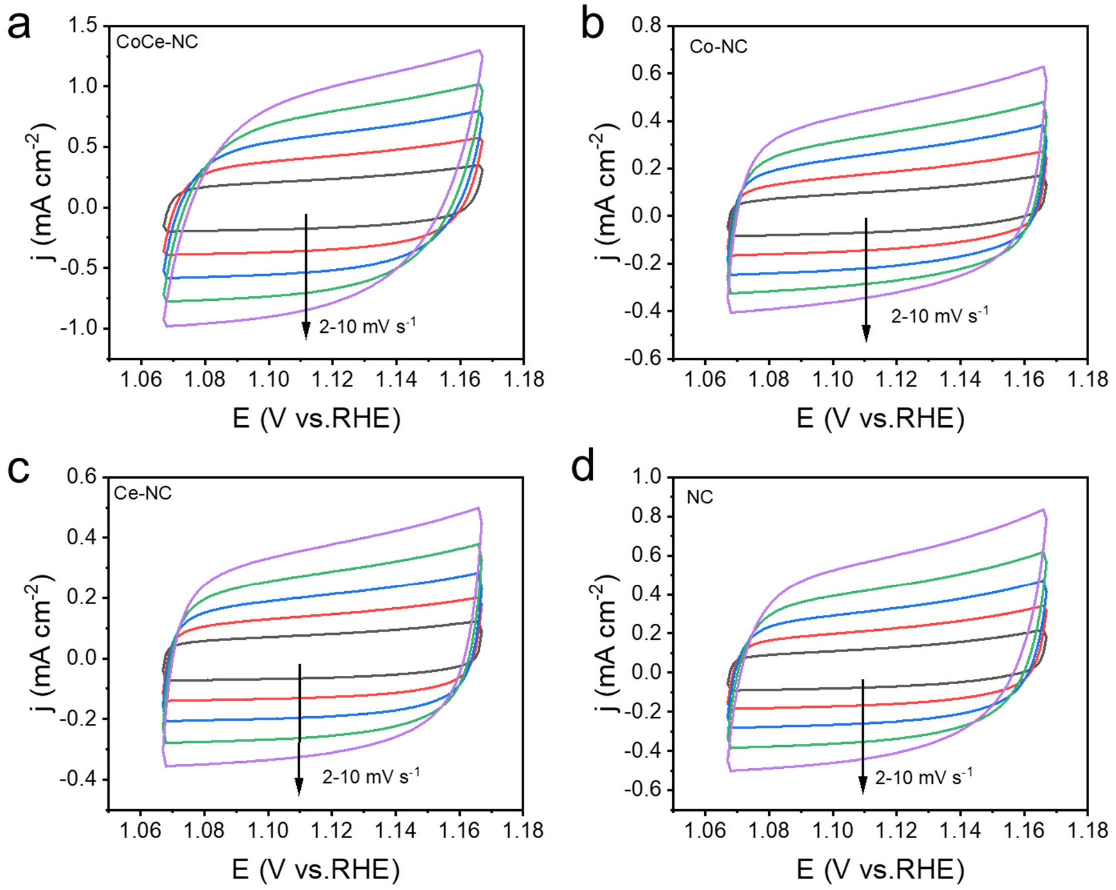

Figure S9. CV of (a)CoCe-NC, (b)Co-NC, (c)Ce-NC, and (d)NC in 0.1 M KOH with different scan rates (10-50 mV s^{-1}) in the non-Faraday interval.

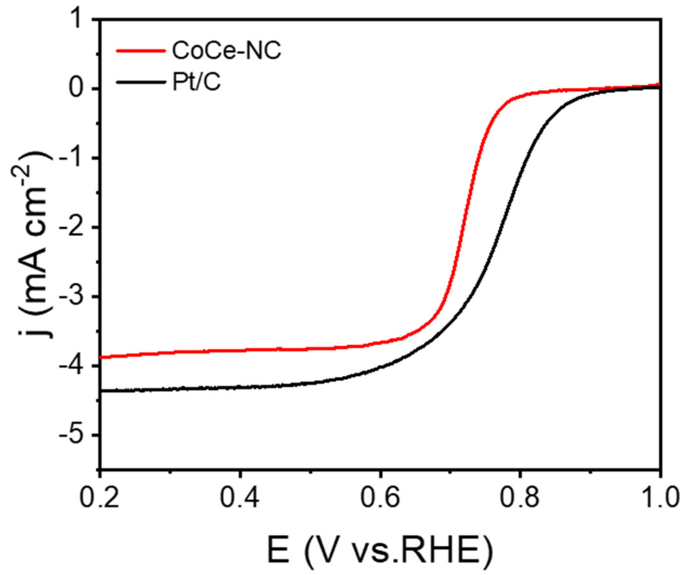

Figure S10. LSV curves of catalysts under 0.5 M H_2SO_4 conditions.

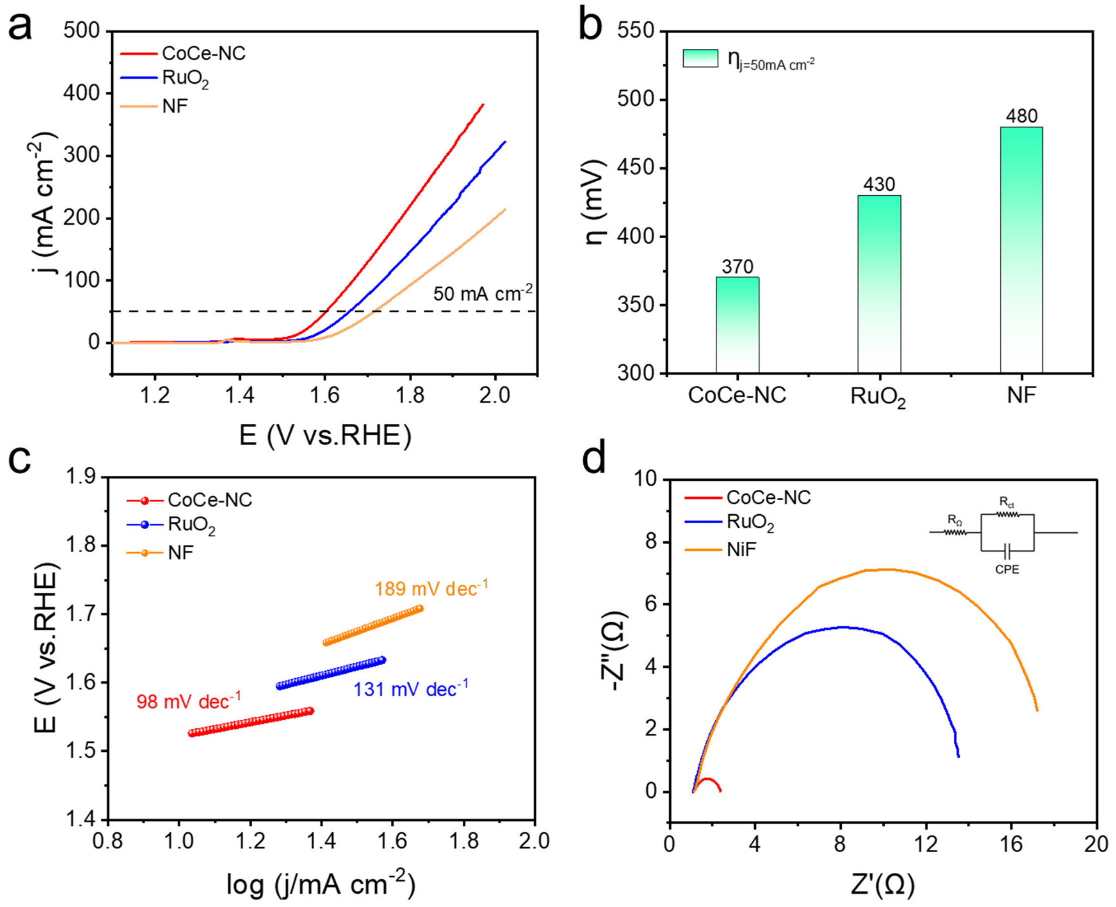

Figure S11. (a) OER LSV curves in 1.0 M KOH. (b) Histogram of Overpotential Distribution. (c) OER tafel slope fitting plots for catalysts. (d) Nyquist plots for catalysts.

a

b

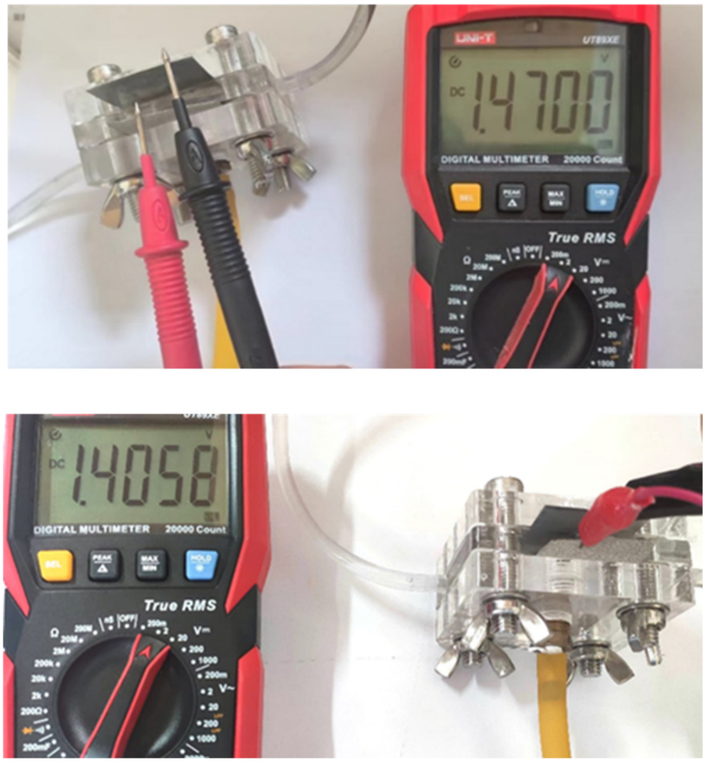

Figure S12. (a) CoCe-NC and (b) Pt/C digital images of output circuit voltage.

Table S1. The content of Co and Ce in the catalyst was obtained by ICP-OES test.

**Catalysts**	**Co (wt.%)**	**Ce (wt.%)**
Co-NC	3.62	0.00
Ce-NC	0.00	0.12
CoCe-NC	2.32	0.09

Table S2. N species and content in the samples were detected by XPS.

**Catalysts**	**Pyridine N (%)**	**Metal-N (%)**	**Pyrrole N (%)**	**Graphite N (%)**	**Oxidation N (%)**
CoCe-NC	29.85	34.57	17.59	14.85	3.14
Co-NC	33.51	29.26	20.47	12.97	3.79

Table S3. Comparison of onset potential, half-wave potential, and limiting current density.

**Catalysts**	**E_{onset} (V vs. RHE)**	**$E_{1/2}$ (V vs. RHE)**	**$J_{limiting}$ (mA cm^{-2})**
CoCe-NC	0.967	0.854	4.52
Co-NC	0.927	0.825	3.67
Ce-NC	0.935	0.833	3.28
NC	0.857	0.774	2.45
Pt/C	0.967	0.847	4.76

Table S4. Comparison of the ORR activity in terms of onset potential and half wave potential for catalyst in this work and the reported non-precious catalysts.

**Catalyst**	**E_{onset} (V vs. RHE)**	**$E_{1/2}$ (V vs. RHE)**	**Reference**
CoCe-NC	0.97	0.86	This Work
Co_3Fe_7/CNs	0.97	0.85	[1]
Co,Fe-HNC-1	0.92	0.85	[2]
F-N/ FeCoNC900	0.97	0.87	[3]
FeCo-N,P-HCS	1.01	0.85	[4]
FeCo DSAs-NCCs	0.99	0.88	[5]
FFCN-MP4000	0.96	0.83	[6]
FB-CoFe-700	0.92	0.82	[7]
CoFeN-NCNTs//CCM	0.92	0.83	[8]
a-MnOx/TiC	0.96	0.8	[9]

Table S5. Comparison of peak power density of catalysts from recent related works.

Catalysis	Peak power density (mW cm^{-2})	Reference
CoCe-NC	202	This Work
FNC/NCS	138	[10]
CoO-TiO_2@NG	146.8	[11]
Co/NHCB	162.2	[12]
ZnS@C-2	120.4	[13]
Co-N/P-C	173.9	[14]
NiFe/$W_{0.3}$C@NC	57.2	[15]
h-$Ti_3C_2T_x$@Co-NCNT-2	160.4	[16]
H-$CoTe_2$/$NiTe_2$@NCBs	166.5	[17]
Pd–Co_3O_4/C	186	[18]
FeCo-NC	146.8	[19]
CoNi-NCNT	180.1	[20]

References

1. Wen, G.-L.; Niu, H.-J.; Feng, J.-J.; Luo, X.; Weng, X.; Wang, A.-J. Well-Dispersed Co3Fe7 Alloy Nanoparticles Wrapped in N-Doped Defect-Rich Carbon Nanosheets as a Highly Efficient and Methanol-Resistant Catalyst for Oxygen-Reduction Reaction. *Journal of Colloid and Interface Science* **2020**, *569*, 277–285, doi:10.1016/j.jcis.2020.02.089.
2. Zhang, Y.; Zhu, T.; Zhong, Q.; Qu, H. Constructing Co/Fe-Nx Dual-Site Catalyst Based on $Co_{0.72}Fe_{0.28}$ Alloy Nanoparticles Anchored on Hollow Hierarchical Porous Carbon Framework for Enhanced Oxygen Reduction Reaction and ZABs. *Journal of Alloys and Compounds* **2023**, *958*, 170447, doi:10.1016/j.jallcom.2023.170447.
3. He, X.; Chang, L.; Wu, H.; Liu, G.; Zhang, Y.; Zhou, A. Design of ZIF-67-Derived Fe, N and F Co-Doped Porous Carbon Material and Evaluation of Its ORR and OER Performance. *Journal of Alloys and Compounds* **2023**, *967*, 171709, doi:10.1016/j.jallcom.2023.171709.
4. Huang, Z.-X.; Wu, D.-H.; Chen, M.-T.; Feng, J.-J.; Wang, A.-J. Confinement Pyrolysis of FeCo Dual-Single Atoms Electrocatalyst for Significantly Boosting Oxygen Reduction Reaction and Rechargeable Zn-Air Battery. *Colloids and Surfaces A: Physicochemical and Engineering Aspects* **2023**, *679*, 132567, doi:10.1016/j.colsurfa.2023.132567.
5. Feng, R.; Ruan, Q.-D.; Feng, J.-J.; Yao, Y.-Q.; Li, L.-M.; Zhang, L.; Wang, A.-J. Facile

Pyrolysis Synthesis of Abundant FeCo Dual-Single Atoms Anchored on N-Doped Carbon Nanocages for Synergistically Boosting Oxygen Reduction Reaction. *Journal of Colloid and Interface Science* **2024**, *654*, 1240–1250, doi:10.1016/j.jcis.2023.10.134.

6. Wu, M.; Xie, J.; Liu, A.; Jia, W.; Cao, Y. Iron Carbide/Nitrogen-Doped Carbon Core-Shell Nanostrctures: Solution-Free Synthesis and Superior Oxygen Reduction Performance. *Journal of Colloid and Interface Science* **2020**, *566*, 194–201, doi:10.1016/j.jcis.2020.01.078.
7. Meng, H.; Pei, S.; Li, H.; Zhang, Y. CoFe/N, S–C Featured with Graphitic Nanoribbons and Multiple CoFe Nanoparticles as Highly Stable and Efficient Electrocatalysts for the Oxygen Reduction Reaction. *ACS Omega* **2021**, *6*, 11059–11067, doi:10.1021/acsomega.1c01024.
8. Zhou, G.; Liu, G.; Liu, X.; Yu, Q.; Mao, H.; Xiao, Z.; Wang, L. 1D/3D Heterogeneous Assembling Body as Trifunctional Electrocatalysts Enabling Zinc–Air Battery and Self-Powered Overall Water Splitting. *Adv Funct Materials* **2022**, *32*, 2107608, doi:10.1002/adfm.202107608.
9. Song, S.; Li, W.; Deng, Y.-P.; Ruan, Y.; Zhang, Y.; Qin, X.; Chen, Z. TiC Supported Amorphous MnOx as Highly Efficient Bifunctional Electrocatalyst for Corrosion Resistant Oxygen Electrode of Zn-Air Batteries. *Nano Energy* **2020**, *67*, 104208, doi:10.1016/j.nanoen.2019.104208.
10. Qing, B.; Liu, Y.; Yang, D.; Yang, M.; Liu, B.; Chen, H.; Li, H. Decrypting Synergy of Alloy & Metal Nanoparticles Within Nitrogen-Doped Carbon Nanosheets for Zn-Air Batteries with Ultralong Cycling Stability. *Small Methods* **2024**, 2401338, doi:10.1002/smtd.202401338.
11. Ding, Q.; Zhang, Q.; Li, B.; Cai, D.; Wu, W. Preparation of Bifunctional Oxygen Evolution Reaction and Oxygen Reduction Reaction Catalyst CoO-TiO_2@NG by High Gravity-Hydrothermal Method for Rechargeable Zn Air Battery. *Journal of Power Sources* **2025**, *625*, 235675, doi:10.1016/j.jpowsour.2024.235675.
12. Wang, F.; Wang, L.; Wang, B.; Jing, Z.; Ding, D.; Yang, X.; Kong, Y.; Dou, J.; Mamoor, M.; Xu, L. Cognate Cobalt Core-Shell Structure Decorated Nitrogen-Doped Hollow Carbon Bowls Triggering Advanced Zinc-Air Battery. *Adv Funct Materials* **2024**, 2415326, doi:10.1002/adfm.202415326.
13. Lin, C.; Yin, Y.; Wang, G.; Cao, X.; Ma, J. Carbon-Coated ZnS as a High-Performance ORR/OER Bifunctional Cathode Catalyst for Zinc-Air Batteries. *International Journal of Hydrogen Energy* **2024**, *93*, 221–228, doi:10.1016/j.ijhydene.2024.10.336.
14. Qi, Y.; Liang, Q.; Song, K.; Zhou, X.; Liu, M.; Li, W.; Liu, F.; Jiang, Z.; Zou, X.; Chen, Z.; et al. Optimizing High-Coordination Shell of Co-Based Single-Atom Catalysts for Efficient ORR and Zinc-Air Batteries. *Journal of Energy Chemistry* **2024**, *95*, 306–314, doi:10.1016/j.jechem.2024.03.049.
15. Jang, E.; Cho, J.; Kim, J.; Kim, J. WC Nanoparticles and NiFe Alloy Co-Encapsulated in N-Doped Carbon Nanocage for Exceptional OER and ORR Bifunctional Electrocatalysis. *Applied Surface Science* **2024**, *663*, 160201, doi:10.1016/j.apsusc.2024.160201.
16. Hao, M.; Li, T.; Lin, L.; Zhang, X.; Huo, C.; Zhang, X.; Liu, X.; Zhu, Y.; Zhang, W. Hollow $Ti_3C_2T_x$ MXene Sphere-Based ZIF-67 Derived Central Radiative Cobalt-Tipped Carbon Nanotubes Electrocatalysts for ORR and OER. *Colloids and Surfaces A: Physicochemical*

and Engineering Aspects **2024**, *688*, 133626, doi:10.1016/j.colsurfa.2024.133626.
17. Liu, M.; Li, Q.; Xiao, X.; Ma, X.; Xu, X.; Yin, Y.; Zhang, B.; Ding, M.; Zou, J.; Jiang, B. $CoTe_2/NiTe_2$ Heterojunction Embedded in N-Doped Hollow Carbon Nanoboxes as High-Efficient ORR/OER Catalyst for Rechargeable Zinc-Air Battery. *Chemical Engineering Journal* **2024**, *486*, 150256, doi:10.1016/j.cej.2024.150256.
18. Akbarian, P.; Eshghi, A.; Asadi, A.; Kheirmand, M. Pd–Co_3O_4/Acetylene Black Nanocomposite as an Efficient and Robust Bifunctional ORR/OER Electrocatalyst for Rechargeable Zinc-Air Batteries. *International Journal of Hydrogen Energy* **2024**, *91*, 1103–1112, doi:10.1016/j.ijhydene.2024.10.225.
19. Zhang, X.; Gao, C.; Li, L.; Yan, X.; Zhang, N.; Bao, J. Fe Based MOF Encapsulating Triethylenediamine Cobalt Complex to Prepare a FeN_3-CoN_3 Dual-Atom Catalyst for Efficient ORR in Zn-Air Batteries. *Journal of Colloid and Interface Science* **2024**, *676*, 871–883, doi:10.1016/j.jcis.2024.07.176.
20. Du, Y.; Zhong, Z.; Shi, Z.; Zhou, L.; Pan, S.; Xu, X.; Liu, Y.; Xiong, D.; Wang, K. Dual-Ligand Engineered CoNi Alloy/N-Doped Carbon Nanotubes Bifunctional ORR/OER Electrocatalyst for Long-Lifespan Rechargeable Zn-Air Batteries. *Journal of Colloid and Interface Science* **2025**, *683*, 631–640, doi:10.1016/j.jcis.2024.12.036.
